# The Origin, Nature and Treatment of Fever, Etc.

**Published:** 1873-08

**Authors:** Ch. Rauschenberg

**Affiliations:** Atlanta, Ga.


ATLANTA

### MEDICAL AND SURGICAL JOURNAL.

VOL. XI.—AUGUST, 1873.—NO. 5.

---

### ORIGINAL COMMUNICATIONS.

---

### THE ORIGIN, NATURE, AND TREATMENT OF FEVER IN THE LIGHT OF MODERN PHYSIOLOGY.

BY CH. RAUSCHENBERG, M.D., ATLANTA, GA.

The changes which have gradually taken place during the last twenty or twenty-five years in the views and conceptions of the medical profession of Europe, and particularly of Germany, in relation to the causation and nature of the febrile process, and the fundamental principles governing its treatment, have as yet—if my intercourse with medical men, and my acquaintance with the current medical literature of this country, enable me to judge correctly—obtained but a very limited recognition on the part of the profession at large in Georgia, and perhaps elsewhere throughout this country, and have not yet exercised any marked influence on the current of thought, and the therapeutical action of medical practitioners generally, in relation to this subject. If this observation is not entirely incorrect, it will explain why I bring this old but important matter, in its new physiological relations and pathological features, to the consideration of the profession.*

* A synopsis of this paper was read before the Georgia Medical Association, at its meeting in this city, last April, but was not published in the Transactions, as the author was unavoidably prevented from finishing it in due time. C. R.

The pathological process commonly called fever has, more than any other one, engaged the general attention of the medical world since the days of Hippocrates. Its name, from the Latin "febris," and the Greek "püretos," *heat*, indicates plainly that the ancients had already recognized unusual heat of the body as its most prominent and most constant symptom, and when Hippocrates advocated the doctrine of critical days and critical phenomena in fevers, as manifestations of the internal process of coction by which the morbid substances were gradually prepared and fitted up for elimination from the system, he already sowed the seed which, two thousand years later, ripened into the views of von Helmont, Sydenham, Stahl, Boœrhave and others, who looked upon fever as an increased activity of the vital process, as a curative effort of nature intended to reëstablish health, or in their own words, as: "*Instrumentum naturæ, quo partes impuras a puris secernat*" (Sydenham); or as "*actum vitatem motorium, secretorium et excretorium mediante quo præsentes quædam noxæ removeantur*" (Stahl.)

It would be an easy matter to fill pages with a recapitulation of the many theories which have since then been developed and entertained by different medical authorities. De Haen, Cullen, Brown, Reil, Pinel, Broussais, Schoenlein, and others, have advanced more or less abstract—Henle, Stilling, Wunderlish, and others, more exact neuropathic—doctrines on the origin of fever, based upon the physiological discoveries of Bell, Müller, and Marshal Hall. A consideration of these or the still later observations and views of Claude Bernard, Ttschetchechin, Traube, etc., on the same subject, seeking to establish, as a cause of fever, a morbid depression of the nerve-centers, regulating the production of caloric within the organism, falls beyond the limits of this paper, which is only intended to embrace those points relating to genesis, nature, and treatment of fever, which have—*as far as its author is able to judge*—the greatest weight of evidence in favor of their correctness and are most generally accepted at the present period of time.

The prominent merit of all pathological investigations of the present day consists in the persistent effort to base all our conceptions upon physiological and anatomical observations and facts, and to define morbid phenomena as perceptible changes of function, and, if possible, as tangible changes of structure of definite elements, parts, or spheres of the system, necessarily resulting from the operations of the laws of life under certain abnormal influences.

When we undertake to analyze the abnormal organic process of life, which we call fever, in its most general sense—not as a specific disease, or species of fever, but as the most frequent result and manifestation of morbid impressions upon the human organization—we should very cautiously distinguish permanent and important from accidental and unimportant, primary from secondary, and coördinate from subordinate occurrences.

*Phenomena* which exist in every case of febrile excitement as primary effects of the cause and coördinate to each other are : *Acute hyperæmia of the capillaries of the entire system, with expansion of their volume, redness and heat of the surface and tissues generally, and a more or less marked general increase of the temperature of the body above the normal standard, connected and seemingly depending upon an increased activity of the heart and arteries.**

These are the constant and the essential pathognomonic marks of fever; but as the degree and uniformity of their development depend, like the normal circulation, upon the quantity and the quality of the blood and the irritability of the nervous system, in each case the production of caloric as well as the degree of vascular excitement and capillary hyperæmia will, in different cases, exhibit different grades of development, and these again will be grouped together in various combinations. In debilitated and anæmic individuals with weak and frequent contractions of the heart, as the normal condition, febrile excitement will often cause only very moderate elevation of temperature, and lessen, for a time, the frequency and increase the force of the heart's action. In extreme cases of that kind, and in the last stages of long-continued fevers, the frequency of the pulse will be very great; but the production of heat, from want of material, so small that the degree of bodily temperature, while it is still relatively higher than it would be without the fever, may not reach the normal temperature of the body, but *without a relative or absolute increase of bodily temperature, fever proper, does not exist, and the very essence of fever consists in an increased metamorphosis of the organic material of the body.*

* Spiess' Pathologische Physiologie. See chapter on Fever.

The disengagement of latent heat in nature takes place, in a large number of chemical processes, in consequence of the mechanical rearrangement of the molecules constituting the substances engaged in action. In men and higher animals, because their organisms consist principally of oxydizable material, and the oxygen of the atmospheric air is in constant and direct contact with them, the production of animal heat is principally, but not exclusively the result of that specific chemical process called oxydation, which takes place partly within the blood, partly within the tissues, simultaneously with their metamorphosis, and in close proximity to the capillaries, which furnish oxygen and nutritive material on the one hand, and remove carbonic acid and waste products on the other.

The question arises now, How is this increased metamorphosis of tissue, this increased oxygenation and combustion, primarily occasioned? Do the first causes of it originate in the blood or in the nervous system, and what is their nature?

The experiments of Billroth,* Bergman† and others, have proven beyond a doubt that the introduction of foreign substances into the blood produces increased oxydation within it and the tissues, or, what is the same, fever. Distilled water, healthy blood, blood serum, the blood of fever patients, pus, putrid substances, pure pepsin, have been used for this purpose, and an increase of the temperature of the test animals, more or less considerable, and of longer or shorter duration, has invariably been recognized after these injections. In traumatic fevers, pyæmia, septicæmia, etc., the presence of foreign substances in the blood has almost become a certainty, and in the contagions, or so-called zymotic or catalytic diseases (measles, scarlatina, variola,) whose specific virus seems to be infinitesimally multiplied during the duration of the disease, like yeast during the process of fermentation, the introduction of a specific poison into the blood as the first cause of the disease can not be denied.

These foreign substances, disturbing the morphological integrity and life of the blood—whether they be malaria or contagion from the outside world, mere molecular detritus, puru-

*Billroth and Pitha, Chirurgie, p. 599, 1865.

†Das putride Gift, die putride Intoxication. Petersburger Med. Zeitschrift Band, 1, 1868.

lent or septic material from a hearth of inflammation, from a tuberculous or scrofulous deposit, from the surface of an unhealthy wound, or the mere stagnating contents of the capillaries of a bruised finger, or abnormally retained secretory and excretory matter—are the primary instigators of the increased oxygenation within the blood and tissues, which we call fever.

Where is the physician who, in course of his medical experience has not arrived at the firm conviction that every functional derangement, interfering—to use the most comprehensive language—with the preparation or resorption of the nutritive, or the elimination of effete materials, consequently with the formation and maintainance of a normally composed blood, is one of the common causes of fever? If we frequently notice in comparatively healthy persons a slight acceleration of the pulse, a flushed countenance, a hot surface, or, in other words, a slight febrile excitement after dinner, in all probability caused by an increased afflux of rich chyle, almost as a physiological process, we can not resist the conclusion that *quantitative and qualitative deviations of the material furnished to the blood for the performance of its physiological functions, or the presence of foreign substances within it, disturbing them, are the principal and primary causes of fever.*

Does this view deserve the reproach of favoring a faulty humoral pathology, which attaches undue importance to the condition of the blood in fever and forgets the influence of the nervous system in its causation?

It appears to the humble writer that this reproach would be just if the blood was a fluid, fully perfected by the process of digestion, undergoing no farther changes but quantitative consumption for the nutrition of the body. This, however, is not the case. Oxygen, new material from the lacteals of the alimentary canal, worn-out matter from the tissues, are continually received by it, and are, under the influences of chemical affinities, engaged—the former, in a process of progressive, the latter, in one of retrograde metamorphosis. Thus the blood undergoes continually a series of complicated but regularly established chemical changes, preparing on the one hand materials for the nutrition and growth of the different tissues, on the other, material for elimination and removal by the different secretory and excretory organs, and simultaneously developing and sustaining its own cell-life, and morphological constituents. Taking into consideration the multiplicity of physiological processes in the system, upon which the supply and preparation, the quantity and quality of the material needed for the normal progress of chemical metamorphosis and blood-life depends, the frequency of blood-changes, from the very slightest degree of physiological abnormity to the highest grades of blood-poisoning, and with them the frequent occurrence of all grades of febrile excitement of shorter or longer duration, is easily understood. Thus increased oxydation within the blood and tissues, "fever," is always primarily caused by a disturbance of the normal blood-life, the normal development of the blood within itself by the introduction or retention of more or less noxious substances within it, which interfere with its morphological or chemical integrity, or both. This is the first and primary occurrence; changed innervation caused by and progressing in direct proportion with this blood-change, the secondary one in all fevers. In idiopathic fevers the noxious substance finds its way into the blood directly from the outside world, and causes primarily a morbid condition of the same; in symptomatic fevers a local pathological condition within the organism—for instance, a local inflammation—furnishes this substance,* and the abnormal state of the blood and the fever are secondary phenomena.

Let us now inquire, *What portion of the nervous system participates principally and primarily, next to the first cause, (the morbid change of the blood) in the further development of the febrile process?*†

General hyperæmia, expansion and fullness of the capillaries, increased temperature of the body, with frequent and often full pulse, the pathological symptoms of the febrile process, do most positively indicate a general excitement of that portion of the nervous system which presides over the action of the heart and blood vessels, and which controls the circulation and regulates the metamorphosis of tissue and the production of animal heat, to-wit: *the ganglia and vaso-motor fibres of the sympathetic system.* By their degree and mode of activity the conditions are created, in the volume of the blood vessels and the

* See The Genesis of Acute and Chronic Inflammation, by Dr. S. Samuel. ATLANTA MEDICAL AND SURGICAL JOURNAL, January, 1873, p. 590.

† Spiess' Pathologische Physiologie, p. 97, etc.

frequency and strength of the contractions of the heart, which restrict or increase the results of the chemical affinity between the free oxygen always present in the blood of the capillaries and the organic tissues susceptible of oxydation.

The ganglionic system consists principally of motor fibres. The sympathetic fibres of the heart, the motory fibres of the stomach and alimentary canal, and also the motory fibres of the blood vessels, excite respectively the muscles of the heart, of the intestines, the smooth muscular fibres of the arteries, and the contractile fibres of the capillary walls in the same manner as the cerebro-spinal motory fibres excite those of the voluntary muscles over which they preside; but they do not receive their impulses for motory action from one general center, like the cerebro-spinal nerves, but generally direct and immediately at the locality where their action becomes manifest. It can not be denied that the action of the heart and arteries can be influenced from the cerebro-spinal centers by the agency of the nerve ganglia, from which the nerves of the heart originate; but the fact that the normal action of the heart takes place independent of the influence of the cerebro-spinal system is just as well established. A frog's heart continues its rythmic action even after having been separated from the body. The action of the smooth muscular fibres of the stomach and intestines, the contractions of the womb, of the bladder, of the contractile ducts of excretory glands, are almost without a doubt dependent upon the mechanical irritation of their ganglionic nerves and nerve-centers alone, by their accumulating contents. So is the contraction of the muscular fibres of the skin excited by the impressions of the cold atmosphere, and the contractions of the capillaries and arteries, and of the heart, by the irritation produced by the living blood which they contain. Oxygenized blood of a certain temperature is the indispensable requisite for the action of the heart and blood vessels in higher animals and in men.

While the anatomical connections of the ganglia amongst themselves and with the cerebro-spinal system, and their mutual influence and dependence upon each other, the existence of localities within the medulla oblongata, spinal marrow, and pneumogastric nerve, which, when irritated or destroyed, produce respectively increased or decreased action of the heart or tone of the vessels, is by no means denied—as an absolute independence of any one portion of the system does not exist in any living organism—*a relative but very marked independence and isolation of the sympathetic nerve and its ganglia from each other, and also from the cerebro-spinal centers,* is claimed as a characteristic feature of this system of nerves, and as an essential element for the correct comprehension of its physiological and pathological manifestations.

If we now inquire how such a general state of excitement of the entire vaso-motor system, as exists in the febrile process, can be brought about, we must necessarily come to the conclusion that an entire province of nerves can only be affected—1. *Either by irritation of its general center and the uniform and simultaneous diffusion of the same to all its peripheric branches; or* 2. *By a uniform and simultaneous irritation of all the branches within their peripheric course and extension.*

One common center of the ganglionic system of nerves, from which all its parts and functions are simultaneously, and in the same manner influenced, has not yet been discovered, and its existence, from many reasons, is quite improbable. The very safety of the organism forbids such an arrangement in a system of nerves, which provides for the supply of the different organs and all the different parts of the body with nutritive material. If such a center existed, or if all the vaso-motor nerves were alike subservient to impressions from the brain and spinal marrow, the nutrition of the different organs and parts of the system, which is exclusively presided over by that system, would be much more subject to general disturbances and not so often confined to single organs.

If the vaso-motor nerves of the organism can not be excited from one common center, the cause of the irritation which produces febrile excitement must be generally diffused throughout the entire organism in such manner that it remains in immediate and permanent contact with all the vaso-motor nerves simultaneously.

The blood alone, and particularly the blood within the capillary system, comes in immediate and permanent contact with the vaso-motor nerves—and therefore only blood deviating from the healthy condition in some manner—and its influence on the peripheric extremities of the vaso-motor nerves can, *primarily,*

occasion that excited condition of the entire system of vaso-motor nerves upon which the febrile process depends. The simultaneous influence of such a morbidly changed blood upon the different ganglionic centers, as well as the centers of the cerebro-spinal system, through which it flows, can not be denied, as many of the different phenomena accompanying different kinds and types of fever, are produced by this influence; but the essential phenomenon of fever—the morbidly exalted organico-chemical process and the subsequent morbid production of caloric and the characteristic changes in the capillary circulation—*are primarily excited by the direct influence of a morbidly altered blood, upon the peripheric vaso-motor nerves.*

The view that fever, although its first cause might be in the blood, could only be caused by the intermediate action of the brain, and particularly the spinal marrow, has been frequently advocated, but not well sustained. Phenomena which originate from the cerebro-spinal axis, bear a very different character in their manifestations from that of fever. They are generally very changeable as to intensity, duration and locality, and do not exhibit the regular uniform character of the febrile process, and indeed the most intense diseases of the substance of the brain and spinal marrow, unless they be inflammations, run their course positively without any fever. *The general, uniform and permanent abnormities of the circulation and production of caloric peculiar to fever can, therefore, only be successfully explained by the existence of a cause within the blood, in continued contact with the peripheric vaso-motor nerves, which stimulates the latter into morbid activity as long as it remains there.*

The heat, the hyperæmia of the capillaries and the increased action of the heart characterizing fever are the immediate consequences of the increased activity of the vaso-motor nervous system, and their degree of manifestation depends upon the quantity of blood, its supply with oxygen, the condition of the structures of the heart and blood vessels, and the irritability of these nerves and the nervous system generally. According to the quality and quantity of the blood and the degree of irritability of the nervous system or the manner in which these different conditions may be grouped together in different individuals, we meet with sthenic and asthenic, erethic and torpid, cases of fever and their different grades and combinations.

But not only the phenomena of the hot stage of fever—if we take a regular paroxysm of intermittent fever as the most perfect and uncomplicated representation of the febrile process—but all others are alike susceptible of rational explanation by this increased action of the vaso-motor system of nerves.

*Every irritation of the vaso-motor nerves causes first a contraction of the blood vessels, a vascular spasm followed by dilatation.* The symptoms of the cold stage are fully explained by this fact. The contractile fibres of the cutaneous tissues, in consequence of the suspension of their normal turgor, the tempory anæmia and loss of caloric connected with it, contract, and therefore the surface of the body first appears cold—so much more so as external atmospheric impressions act, under these circumstances, as additional irritants. Thus the manifestations of the increased action of the vaso-motor nerves is counteracted for a time, and the symptoms at the commencement of a febrile paroxysm apparently contradict the idea of increased vaso-motor action and increased production of heat. As this contraction of the skin, under the continued impression of the fever-cause, yields, and as its capillaries cease to be contracted, the skin receives gradually more and more blood, the action of the heart becomes more and more free and unimpeded, the skin more and more hyperæmic, and the increased metamorphosis and increased production of heat are more and more developed, and become more distinctly perceptible.

These phenomena, however, do not reach the highest degree of intensity as long as the existing cause of irritation of the vaso-motor nerves prompts the arteries to increased contraction. This contraction, so perceptible in the tense pulse of the hot stage, is only gradually overcome by the active hyperæmia, the increased impetus of the heart, the increased organico-chemical process, and the increased metamorphosis in the tissues. In consequence of the latter, an abnormal consumption of the water of the solid and liquid constituents of the body is established, and with it the thirst so characteristic in fever. Again, in consequence of this deficiency of water, and of the still imperfect relaxation and expansion of the capillaries, the secretions and excretions continue to appear restricted; while, in reality, with the increased metamorphosis and combustion in the tissues, they have also increased, but their products are retained within the blood of the different organs.

*With the decrease of the exalted vaso-motor action, the hyperæmia of the capillaries reaches its highest degree*, because, while the stormy action of the heart ceases only gradually, no obstacle obstructs, at the periphery of the circulation, any longer the wave of the blood current, and the exalted organico-chemical process in the tissues still continues awhile, in gradually decreasing strength, to attract and to consume more than the normal quantity of blood.

With this stage of the fever, when the irritation of the vaso-motor nerves has ceased, and an entire relaxation of the walls of the capillaries and arteries has been established, the secretions and excretions show necessarily a considerable increase in quantity generally, as well as in the relative amount of their characteristic constituents—for instance, urea in the urine—because now the products of the febrile metamorphosis are removed from the blood by the different secretory and excretory organs.

Thus the name "sweating stage," for this period of fevers is certainly not very proper, as all excretory organs are in the same physiological condition, and engaged in the same manner as the excretory apparatus of the skin, whose action, in all probability, is not of the same general importance in the removal of the febrile products as that of the kidneys.

A more rational, uniform, and harmonious explanation of all the phenomena of fever in their regular course of occurrence as the theory advanced in the preceding pages furnishes, the writer has not been able to find anywhere else, but in the admirable work of Dr. G. A. Spiess, Physiological Pathology,* from which the above views were principally taken, but which, unfortunately, with its rich treasures of pathological truths on all the subjects of medicine, has not yet been translated into the English language.

Many other theories in relation to the agency of the nervous system in the production of the febrile process, very different from the one advocated in the preceding pages, have been advanced. They are all based upon isolated physiological observ-

* Physiologische Pathologie, von Dr. G. A. Spiess.

ations appertaining to the influences of certain portions of the nervous system on the circulation of the blood. It is evident that this, with its many different phenomena—such as frequency of pulse, degree of blood pressure in the heart and arteries, velocity of the blood current itself—is dependent upon various more or less important nerve influences, emanating not only from the sympathetic, but also from the cerebro-spinal portions of the nervous system. While the ganglionic centers and vaso-motor nerve fibres preside primarily and directly over the action of the muscular fibres of these organs, and while, without the presence of normal blood within the capillaries of the heart and the blood vessels, as the peripheric stimulants of their ganglia and vaso-motor nerve fibres, no cerebro-spinal influences can affect these organs, the facts that division or paralysis of the vagus, or an irritation of the cervical portion of the sympathetic or medulla oblongata, exercise a controlling influence over the action of the heart and arteries—in the former case increasing, in the latter one decreasing, the frequency of their contractions — and that irritation of the medulla oblongata causes an increase in the tonicity and an abnormal contraction of the finer arteries—hence backward blood-pressure in the larger ones, and in the heart, etc.—are too well established by numerous experiments to be denied.

They only demonstrate the great complicity of the process of blood-circulation, and therefore the fact that various nerve influences may, in the course of the development of the febrile process become successively instrumental in fully establishing or modifying this morbid condition; but they do not furnish a more rational basis for the interpretation of its morbid phenomena, nor disprove the fact that the vast system of vaso-motor nerves, in immediate contact with all blood vessels, is the first recipient of all morbid impressions arising within its most natural physiological stimulant, *the blood*, and that the febrile process takes its origin prominently and primarily within *that* portion of the nervous system, affecting the cerobro-spinal center only secondarily.

(*To be Continued.*)

